# Serum Th1 and Th17 related cytokines and autoantibodies in patients with Posner-Schlossman syndrome

**DOI:** 10.1371/journal.pone.0175519

**Published:** 2017-04-06

**Authors:** Jun Zhao, Wenchieh Chen, Xiaosheng Huang, Shiming Peng, Tianhui Zhu, Zhihui Deng, Ping Liang, Hui Chang, Bao Jian Fan

**Affiliations:** 1 Shenzhen Key Laboratory of Ophthalmology, Shenzhen Eye Hospital Affiliated to Jinan University, Shenzhen, Guangdong, China; 2 School of Ophthalmology & Optometry Affiliated to Shenzhen University, Shenzhen, Guangdong, China; 3 Immunogenetics Laboratory, Shenzhen Blood Center, Shenzhen, Guangdong, China; 4 Department of Ophthalmology, Harvard Medical School, Massachusetts Eye and Ear Infirmary, Boston, Massachusetts, United States of America; Oregon Health and Science University, UNITED STATES

## Abstract

Posner-Schlossman syndrome (PSS) shares some clinical features with uveitis and open angle glaucoma. Cytokines and autoantibodies have been associated with uveitis and open angle glaucoma. However, the role of serum cytokines and autoantibodies in the pathogenesis of PSS remains unknown. This study aimed to evaluate the associations of type 1 T helper (Th1) and Th17 related cytokines and autoantibodies with PSS. Peripheral blood serum samples were collected from 81 patients with PSS and 97 gender- and age-matched healthy blood donors. Th1 and Th17 related cytokines, including interleukin-1β (IL-1β), IL-12, tumor necrosis factor-α (TNF-α), interferon- γ (IFN-γ), IL-6 and IL-17, and glucose-6-phosphate isomerase (GPI) were determined by double antibody sandwich ELISA. Anti-nuclear antibody (ANA), anti-keratin antibody (AKA) and anti-neutrophil cytoplasmic antibody (ANCA) were detected by indirect immunofluorescence assay. Anti-cardiolipin antibody (ACA)-IgG, ACA-IgM, ACA-IgA, anti-double stranded DNA (anti-dsDNA) and anti-cyclic citrullinated peptide antibody (anti-CCP) were detected by indirect ELISA. Serum levels of IL-1β, IL-12 and IL-6 in PSS patients were significantly lower than those in controls (*P* < 0.003), and these associations survived the Bonferroni correction (*P*_*c*_ < 0.018). There was no significant difference in serum levels of TNF-α, IFN-γ and IL-17 between the PSS and control groups (*P*_*c*_ > 0.12). Positive rate of serum anti-dsDNA in PSS patients was significantly higher than that in the control group (*P* = 0.002, *P*_*c*_ = 0.018), while positive rates of serum ANA, AKA, ANCA, ACA-IgG, ACA-IgM, ACA-IgA, GPI and anti-CCP in the PSS group were not significantly different from those in the control group (*P*_*c*_ > 0.09). These results suggest that anti-dsDNA may contribute to the pathogenesis of PSS, while Th1 and Th17 related cytokines and other autoantibodies may not be major contributors to PSS.

## Introduction

Posner-Schlossman syndrome (PSS) was first described in detail by Posner and Schlossman in 1948 [1]. PSS has some clinical manifestations similar to glaucoma and uveitis, such as high intraocular pressure (IOP) and keratic precipitates (KPs). Little attention has previously been paid to PSS, because some patients can usually be self-healing or misdiagnosed. In recent years, PSS was not only found to be often recurrent and difficult to cure, but also could cause irreversible visual impairment or even blindness in some patients [2]. The etiology of PSS remains unclear. Pathogenic microbial infection mainly caused by cytomegalovirus (CMV) has been proposed as the initial and leading cause of PSS [3,4]. In addition, cytokine, allelic heterogenicity and vascular endothelial dysfunction can all contribute to the pathogenesis of PSS [4–7].

Cytokines are mainly secreted by helper T cells and have many biological roles in regulating cell growth, differentiation, inflammation, immune response and tissue repair. According to the cytokines secreted by helper T cells and their functions, helper T cells can be classified into several subsets such as type 1 T helper (Th1) and type 17 T helper (Th17). Th1 and Th17 cells are the main inflammatory cell subsets leading to autoimmune uveitis. Major Th1 and Th17 related cytokines include interleukin-1β (IL-1β), IL-12, tumor necrosis factor-α (TNF-α), interferon- γ (IFN-γ), IL-6 and IL-17. Recent studies have suggested that cytokines may be involved in the pathogenesis of ocular diseases such as age-related macular degeneration, diabetic retinopathy and dry eye [8–10]. Th1 cytokines may contribute to the inflammatory response in patients with anterior uveitis, and Th17 cytokines may be involved in the pathogenesis of idiopathic uveitis [11]. Th1 cytokine abnormalities in serum and iris have been associated with the pathogenesis of primary open-angle glaucoma (POAG) [12,13]. Intriguingly, Li and coworkers reported that aqueous IL-2, IL-12, TNF-α and IFN-α levels were significantly lower in PSS patients than cataract patients [5]. However, this study has some limitations, such as the small sample sizes (53 PSS patients and 23 cataract controls), the significant differences in gender and age between cases and controls, and the use of cataract patients as controls. In addition, the relationship between serum Th1 and Th17 related cytokines and PSS has not yet been reported.

Autoantibodies refer to immunoglobulins against self-antigens located within cells, on cell surfaces or outside cells of the body. Autoantibodies can produce an acquired immune response with the body’s own components, which is the main cause of autoimmune diseases, such as systemic lupus erythematosus, rheumatoid arthritis and systemic vasculitis. Autoantibodies have been associated with open angle glaucoma [14]. However, the relationship between autoantibodies and PSS has been poorly investigated. One preliminary study of 12 PSS patients revealed that one patient (8.3%) was anti-nuclear antibody (ANA) positive and two patients (16.7%) were anti-cardiolipin antibody (ACA) positive, which were not significantly different from normal controls (n = 15) [7]. Further studies with larger samples are required to confirm these preliminary findings.

Since PSS shares some clinical characteristics of uveitis and open angle glaucoma, and cytokines and autoantibodies have been associated with uveitis and open angle glaucoma, we hypothesized that Th1 and Th17 related cytokines and autoantibodies might be involved in the pathogenesis of PSS. In the present study, we evaluated the associations of serum Th1 and Th17 related cytokines and serum autoantibodies with PSS in a sample of 81 patients with PSS and 97 gender- and age-matched healthy blood donors. We found that serum levels of IL-1β, IL-12 and IL-6 were significantly lower in PSS patients than normal controls, while serum anti-double stranded DNA (anti-dsDNA) had a significantly higher positive rate in PSS patients than controls.

## Methods

### Patients and controls

Eighty-one patients with PSS attending the Clinic of Shenzhen Eye Hospital were enrolled between 2013 and 2015. The diagnosis of PSS was based on the following criteria [1,6,7,15]: 1) unilateral recurrent transient episodes of elevated IOP greater than 21 mmHg with duration of attack varying from a few hours to several weeks. IOP may reach 40 mmHg or more, and spontaneous remission often occurs; 2) mild cyclitis: a few white KPs accumulating in the corneal endothelium observed in the anterior chamber; 3) a slight decrease in vision, no visual field defect and normal optic disc at the early stage; 4) open iridocorneal angle without peripheral anterior synechia. According to previous medical records, patients who had a history of ophthalmic CMV, herpes simplex virus (HSV) or hepatitis B virus (HBV) infection, a history of ocular trauma, ocular laser treatment, or ophthalmic surgery, and a history of autoimmune disease or tumor were excluded from this study [7]. The disease duration for patients with PSS ranged from 8 days to 20 days, with an average of 11.1 (±3.7) days. The intermittent period varied between 2 months and 15 months, with an average of 5.3 (±3.4) months. All patients were in an active disease status who were either a first attack or at the early stage of a return visit (i.e., within 1–3 days). After collecting peripheral blood samples, the patients were treated with topical steroids to control the inflammation and with IOP-lowering medications or filtration surgery to control the IOP.

Ninety-seven healthy volunteer blood donors from the Shenzhen Blood Center were recruited as controls. All control subjects had normal IOP and optic disc, no KPs, no history of any eye diseases, and no history of ophthalmic CMV, HSV or HBV according to previous medical records. The control group was matched in age and gender with the patient group. There was no significant difference in age and gender between the two groups (*P* > 0.22, Table 1). Significantly lower IOP was observed in controls than patients with PSS (*P* < 0.001, Table 1). All patients and controls were self-reported southern Han Chinese.

**Table 1:** Demographic and clinical features of PSS patients and controls.

Feature	PSS (n = 81)	Controls (n = 97)	*P*-value
**Sex (M/F)**	41/40	55/42	0.33^a^
**Age, mean (SD), year**	43.2 (12.4)	41.3 (8.7)	0.22^b^
**IOP, mean (SD), mmHg**	41.7 (7.1)	14.0 (3.5)	< 0.001^b^

Abbreviation: PSS, Posner-Schlossman syndrome; M, male; F, female; SD, standard deviation; IOP, intraocular pressure.

^a^Chi-squared test.

^b^Independent samples t-test.

The study protocol was in accordance with the tenets of the Declaration of Helsinki and approved by the local institutional ethnics committee of Shenzhen Eye Hospital. Written informed consent was obtained from all study subjects.

### Sample collection

Peripheral blood samples were collected in procoagulant drying tubes and stored at room temperature for 1 hr. The samples were centrifuged at 3,000 rpm for 5 min. Serum was aliquoted into Eppendorf tubes, and stored at -80°C before use.

### Detection of CMV-IgG and CMV-IgM by indirect ELISA

Serum samples were diluted 1:100 and 1:50 for detection of CMV-IgG and CMV-IgM respectively. Diluted samples were added to the microplate wells and incubated at 37°C for 0.5 hr according to the manufacturer’s instructions (ELISA kit 20150824, Shenzhen Boca Biotechnology Co., Ltd., Shenzhen, China). The HRP labeled antibody was then added and incubated at 37°C for 0.5 hr. TMB color reaction solution was added to each well and incubated for 10 min. The reaction was terminated by adding stop solution. The OD value of each well at the wavelength of 450 nm was measured by a microplate reader (Model 680, Bio-Rad Laboratories Inc., Japan). The results were considered positive if the OD values of the samples were equal or greater than the OD value of negative control times 2.1, otherwise the results were considered negative. Positive results were confirmed by repeating the detections using the same method. CMV(+) indicates positive for CMV-IgG and/or CMV-IgM, while CMV(-) means negative for both CMV-IgG and CMV-IgM.

### Determination of IL-1β, IL-12, TNF-α, IFN-γ, IL-6 and IL-17 by double antibody sandwich ELISA

Serum samples were diluted 1:5 and added into the microplate wells and incubated at 37°C for 0.5 hr according to the manufacturer’s instructions (Shanghai Enzyme-linked Biotechnology Co., Ltd., Shanghai, China). The horseradish peroxidase (HRP) labeled antibody was then added and incubated at 37°C for 0.5 hr. TMB (3,3’,5,5’-tetramethylbenzidine) color reaction solution was added to each well and incubated for 15 min. The reaction was terminated by adding stop solution. The absorbance (A) at 450 nm was measured with a microplate reader (Model 680, Bio-Rad Laboratories Inc., Japan). The serum levels of cytokines were calculated based on the A values and dilution factors of the samples. The limit of detection was 5–200 ng/L, 5–300 ng/L, 100–2000 ng/L, 50–2000 ng/L, 4–100 ng/L and 3.5–100 ng/L for IL-1β, IL-12, TNF-α, IFN-γ, IL-6 and IL-17, respectively.

### Detection of serum ANA, Anti-Keratin Antibody (AKA) and Anti-Neutrophil Cytoplasmic Antibody (ANCA) by Indirect Immunofluorescence assay (IIF)

Serum samples were diluted 1:100 for detection of ANA and 1:10 for detection of AKA and ANCA. According to the manufacturer’s instructions (Euroimmun Medizinische Labordiagnostika AG, Lubeck, Germany), diluted samples were fixed on the reaction zones of specific biological slides and incubated for 0.5 hr. Human HEp-2 cells and monkey liver were used for detection of ANA; rat oesophagus was used for detection of AKA; and monkey liver and human granulocyte were used for detection of ANCA. The fluorescent-labeled secondary antibody was added onto the slides and incubated for 0.5 hr in the dark. The slides were then mounted with glycerol and observed under fluorescence microscope (EUROStar III Plus, Euroimmun Medizinische Labordiagnostika AG).

IIF positive was determined based on specific fluorescence pattern and antibody titer [16]. Specific fluorescence pattern must be consistent with the positive control serum. For ANA, fluorescence intensity was scored in four categories ranging from 0 to +++, and staining patterns were classified as “homogeneous”, “fine speckled”, “coarse speckled”, “nucleolar”, and “centromere”. For AKA, positive samples demonstrated distinct laminar or speckled fluorescent staining of the superficial layer (stratum corneum) of the rat oesophagus epithelium. For ANCA, fluorescence patterns were classified as cytoplasmic (cANCA) when a diffuse granular cytoplasmic staining was evenly distributed throughout the neutrophil cytoplasm, and as perinuclear (pANCA) when a perinuclear smooth banded fluorescence pattern was observed in the neutrophils. The antibody titers ≥ 1:100 was considered positive for ANA, and the antibody titers ≥ 1:10 were considered positive for AKA and ANCA.

### Detection of serum ACA-IgG, ACA-IgM, ACA-IgA, anti-dsDNA and anti-Cyclic Citrullinated Peptide antibody (anti-CCP) by indirect ELISA and Glucose-6-Phosphate Isomerase (GPI) by double antibody sandwich ELISA

Serum samples were diluted 1:101, 1:41, 1:101, 1:201, 1:50 and 1:1 for detection of ACA-IgG, ACA-IgM, ACA-IgA, anti-dsDNA, anti-CCP and GPI, respectively. Diluted samples were added into the microplate wells and incubated at 37°C for 0.5 hr according to the manufacturer’s instructions (Euroimmun Medizinische Labordiagnostika AG). The HRP labeled antibody was then added and incubated at 37°C for 0.5 hr. TMB color reaction solution was added to each well and incubated for 10 min. The reaction was terminated by adding stop solution. The absorbance (A) at 450 nm was measured with a microplate reader (Model 680, Bio-Rad Laboratories Inc., Japan). The results were considered positive if the A values of the samples were higher than the standard positive control (i.e., 0.8 for ACA-IgG, ACA-IgM and ACA-IgA and 100 IU/ml for anti-dsDNA), otherwise the results were considered negative. The limit of detection was 0–800 IU/ml for anti-dsDNA.

Serum GPI was detected by double antibody sandwich ELISA as described above. The results were considered positive if the A values of the samples were higher than the standard positive control (i.e., 0.20 mg/L). The limit of detection was 0–4 mg/L.

### Statistical analysis

SPSS 20.0 statistical software (SPSS Inc., Chicago) was used for statistical analysis. Independent samples t-test was used to compare the differences in age and IOP between patients and controls. Chi-squared test or Fisher’s exact test was used to compare the differences in gender and serum positive rates of CMV and autoantibodies between the two groups. Since serum levels of Th1 and Th17 related cytokines were not consistent with the normal distribution as suggested by Shapiro-Wilk test, the data were presented as median (Q1, Q3) and Mann-Whitney U test was used to compare the differences between the two groups. Serum mean concentrations of anti-dsDNA were compared between PSS patients and controls using Mann-Whitney U test. Because no significant difference in individual cytokines and autoantibodies was observed between CMV(+) and CMV(-) PSS groups, the CMV(+) and CMV(-) PSS groups were then pooled and compared to controls. Multiple comparisons were corrected using the Bonferroni method and the corrected *P* value (*P*_*c*_) was calculated by multiplying the *P* value with the number of tests performed. *P*_*c*_ < 0.05 was considered statistically significant.

## Results

### Comparison of serum positive rate of CMV-IgG and CMV-IgM between PSS patients and controls

Among 81 patients with PSS, 10 (12.4%) patients were positive for CMV-IgG only, 6 (7.4%) patients were positive for CMV-IgM only, and 8 (9.9%) patients were positive for both CMV-IgG and CMV-IgM. Of 97 normal controls, 4 (4.1%) were positive for CMV-IgG only, and none was positive for CMV-IgM. PSS patients had significantly higher infection rate of CMV than normal controls (29.6% *vs*. 4.1%, *P* < 0.0001; Table 2).

**Table 2:** Comparison of serum positive rate of CMV-IgG and CMV-IgM between PSS patients and controls.

	PSS (n = 81)	Controls (n = 97)	*P*-value^b^
**CMV-IgG(+) only**	10 (12.4)	4 (4.1)	0.04
**CMV-IgM(+) only**	6 (7.4)	0 (0.0)	0.008
**Both CMV-IgG(+) and CMV-IgM(+)**	8 (9.9)	0 (0.0)	0.002
**CMV(+)**^a^	24 (29.6)	4 (4.1)	<0.0001

Abbreviation: CMV, cytomegalovirus; PSS, Posner-Schlossman syndrome.

Data were presented as n (%).

^a^CMV-IgG(+) and/or CMV-IgM(+).

^b^Chi-squared test or Fisher’s exact test.

### Comparison of serum levels of Th1 and Th17 related cytokines between PSS patients and controls

No significant difference in serum levels of all 6 cytokines was observed between CMV(+) and CMV(-) PSS groups (*P* > 0.10; Table 3). Serum levels of IL-1β, IL-12 and IL-6 were significantly lower in PSS patients than controls (*P* < 0.003), and these associations survived the Bonferroni correction (*P*_*c*_ < 0.018; Table 3). No significant difference in serum levels of TNF-α, IFN-γ and IL-17 was found between the patient and control groups (*P*_*c*_ > 0.12; Table 3).

**Table 3:** Comparison of serum levels of Th1 and Th17 related cytokines between PSS patients and controls.

Group	N	IL-1β	IL-12	TNF-α	IFN-γ	IL-6	IL-17
**CMV(+) PSS**	24	37.5 (21.2, 61.3)	77.5 (44.5, 112.4)	524.5 (401.1, 792.3)	564.4 (404.4, 731.9)	23.5 (19.0, 49.5)	27.0 (19.3, 43.9)
**CMV(-) PSS**	57	50.9 (30.6, 110.4)	84.6 (42.1, 167.7)	566.7 (421.2, 1185.4)	643.7 (447.0, 1263.6)	25.4 (19.9, 55.9)	29.4 (21.4, 60.9)
**Control**	97	67.1 (47.4, 104.7)	98.6 (63.7, 178.4)	694.8 (491.2, 1222.5)	638.8 (421.8, 1232.8)	34.6 (25.4, 58.0)	32.6 (22.4, 63.0)
***P***^a^		0.10	0.45	0.36	0.32	0.59	0.46
***P***^b^		0.001	0.003	0.02	0.55	<0.0005	0.12
***P***_**c**_^c^		**0.006**	**0.018**	0.12	1.00	**<0.003**	1.00

Abbreviation: Th1, type 1 T helper; Th17, type 17 T helper; PSS, Posner-Schlossman syndrome; IL, interleukin; TNF-α, tumor necrosis factor-α; IFN-γ, interferon- γ; *P*_c,_ Bonferroni corrected *P* value for multiple comparisons.

Data were presented as median (Q1, Q3) (ng/L).

^a^*P* value from Mann-Whitney U test for difference in cytokine levels between CMV(+) and CMV(-) PSS groups.

^b^*P* value from Mann-Whitney U test for difference in cytokine levels between PSS group [including both CMV(+) and CMV(-)] and controls.

^c^Bonferroni corrected *P* value for *P*^b^.

### Comparison of serum levels of autoantibodies between PSS patients and controls

No significant difference in serum positive rates of all 9 autoantibodies was observed between CMV(+) and CMV(-) PSS groups (*P* > 0.23; Table 4). Serum positive rate of anti-dsDNA was significantly higher in the PSS group than the control group (*P* = 0.002, *P*_*c*_ = 0.018; Table 4). Serum mean concentration of anti-dsDNA was significantly higher in PSS patients than controls (26.2 IU/ml *vs*. 8.7 IU/ml; *P* = 0.03). However, this difference did not survive the Bonferroni correction for multiple comparisons (*P*_*c*_ = 0.29). There was no significant difference in positive rates of ANA, AKA, ANCA, ACA-IgG, ACA-IgM, ACA-IgA, GPI and anti-CCP between the two groups (*P*_*c*_ > 0.09; Table 4).

**Table 4:** Comparison of serum positive rates of autoantibodies between PSS patients and controls.

Group	N	ANA	anti-dsDNA	AKA	ANCA	ACA-IgG	ACA-IgM	ACA-IgA	GPI	anti-CCP
**CMV(+) PSS**	24	4 (16.7)	4 (16.7)	1 (4.2)	0 (0.0)	0 (0.0)	0 (0.0)	2 (8.3)	2 (8.3)	1 (4.2)
**CMV(-) PSS**	57	10 (17.5)	6 (10.5)	2 (3.5)	0 (0.0)	2 (3.5)	4 (7.0)	5 (8.8)	10 (17.5)	1 (1.8)
**Control**	97	11 (11.3)	1 (1.0)	2 (2.1)	0 (0.0)	0 (0.0)	4 (4.1)	3 (3.1)	4 (4.1)	3 (3.1)
***P***^a^		1.00	0.69	0.89	N.A.	0.23	0.44	1.00	0.47	0.54
***P***^b^		0.26	0.002	0.84	N.A.	0.08	1.00	0.20	0.01	1.00
***P***_**c**_^c^		1.00	**0.018**	1.00	N.A.	0.72	1.00	1.00	0.09	1.00

Abbreviation: ANA, anti-nuclear antibody; anti-dsDNA, anti-double stranded DNA; AKA, anti-keratin antibody; ANCA, anti-neutrophil cytoplasmic antibody; ACA, anti-cardiolipin antibody; Ig, immunoglobulin; GPI, glucose-6-phosphate isomerase; anti-CCP, anti-cyclic citrullinated peptide antibody; *P*_c,_ Bonferroni corrected *P* value for multiple comparisons; N.A., not available.

Data were presented as n (%).

^a^*P* value from chi-squared test or Fisher’s exact test for difference in positive rates of autoantibodies between CMV(+) and CMV(-) PSS groups.

^b^*P* value from chi-squared test or Fisher’s exact test for difference in positive rates of autoantibodies between PSS group [including both CMV(+) and CMV(-)] and controls.

^c^Bonferroni corrected *P* value for *P*^b^.

## Discussion

In the present study, to elucidate the role of serum cytokines and autoantibodies in the pathogenesis of PSS, we compared serum levels of major Th1 and Th17 related cytokines (i.e., IL-1β, IL-12, TNF-α, IFN-γ, IL-6 and IL-17) and serum positive rates of autoantibodies (i.e., ANA, anti-dsDNA, AKA, ANCA, ACA-IgG, ACA-IgM, ACA-IgA, GPI and anti-CCP) between PSS patients and normal controls. The primary data were shown in S1 Table. To the best of our knowledge, this is the first report to evaluate the association of serum cytokines with PSS. In view of the important role of CMV in the pathogenesis of PSS [3,4], CMV-IgG and CMV-IgM were further detected in all PSS patients and controls by indirect ELISA. However, since a history of ophthalmic CMV infection had been excluded from both PSS patients and controls by examining their previous medical records, the CMV infection rates of PSS patients and controls were lower than expected in the general population [17].

**Fig 1:**
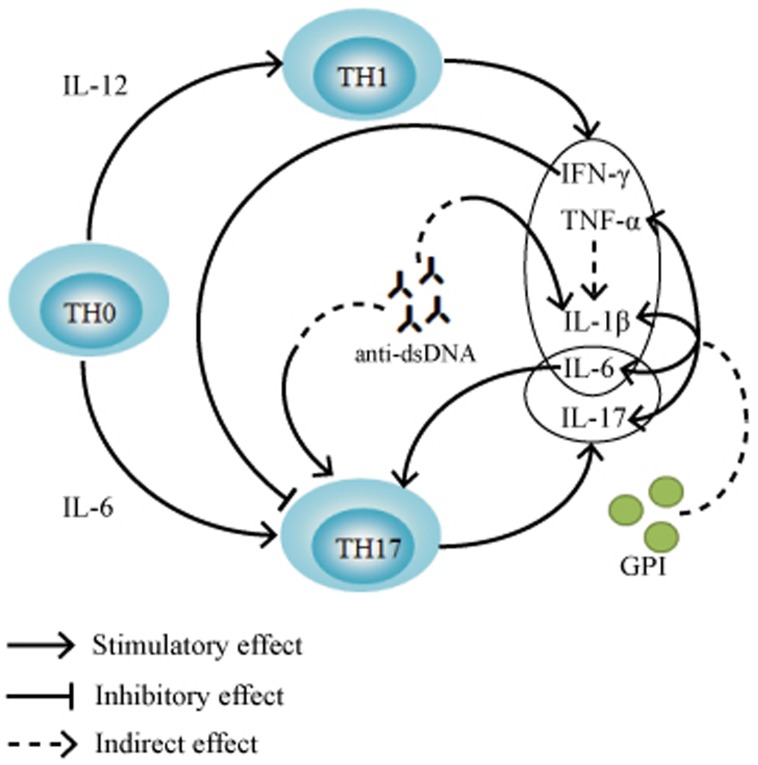
Diagram of the network on interactions among the Th1 and Th17 related cytokines, anti-double stranded DNA (anti-dsDNA) and Glucose-6-Phosphate Isomerase (GPI). Abbreviations: IL, interleukin; TNF-α, tumor necrosis factor-α; IFN-γ, interferon- γ. Arrows indicate stimulatory effect, bar-headed line indicates inhibitory effect and dashed arrows indicate indirect effect.

IL-1 is an early inflammatory cytokine with the ability to regulate immune and inflammatory responses in vivo. Since IL-1α is mainly present in the cell and IL-1β is mainly present in the body fluid, the biological effects of IL-1 are mainly mediated by IL-1β [18]. Fleisher et al. reported that IL-1β could stimulate the production of IL-6 (Fig 1), leading to intraocular inflammation in uveitis [19]. In the present study, we found that serum IL-1β levels in PSS patients were significantly lower than those in normal controls (Table 3). Intriguingly, Li et al. also reported that aqueous IL-1β levels in patients with PSS were lower than those in cataract controls although the difference did not reach statistical significance possibly due to the small sample sizes [5]. These findings indicate that IL-1β might not be the main cytokine responsible for the inflammatory response in PSS.

IL-12 has biological effects such as stimulating the development of Th1 cells and stimulating NK cells and T cells to produce IFN-γ (Fig 1). IL-12 is an important factor in regulation of innate and adaptive immunity [20]. Tarrant et al. reported that increased serum IL-12 could promote the production of IFN-γ and nitric oxide, and further enhance the inflammatory response of autoimmune uveitis [21]. Yokoi et al. reported that anti-IL-12 treatment could inhibit the development of Th1 cells in the animal model of autoimmune uveitis, so as to control the inflammatory response of autoimmune uveitis [22]. In this study, we found that serum IL-12 levels were significantly lower in PSS patients than normal controls (Table 3). Our findings were consistent with the results of Li et al. who found significant decrease of aqueous IL-12 levels in PSS patients than cataract controls [5]. Taken together, these findings suggest that the development of Th1 cells might be inhibited in PSS patients.

IL-6 can be induced by cytokines such as IFN-γ, TNF-α and IL-1β (Fig 1), which is an important cytokine that causes inflammatory response of uveitis [19]. IL-6 can induce the differentiation of T cells into Thl7 cells, and the induced Th17 cells can secrete IL-6, thus forming a positive feedback effect (Fig 1) [23,24]. Experiments in mouse models have shown that IL-6 in aqueous humor is mainly produced through the secretion by the iris [25]. In the present study, we found that serum IL-6 levels were significantly lower in PSS patients than normal controls (Table 3), suggesting that decreased serum IL-6 concentration in PSS may be associated with the characteristics of open-angle glaucoma in PSS. Of note, our results were inconsistent with the findings of significantly increased aqueous IL-6 levels in patients with PSS in a study by Li et al. [5]. These discrepancies may be due to several differences between the two studies. First, the sample sizes in this study (81 PSS patients and 97 normal controls) were larger than those in the study by Li et al. (53 PSS patients and 23 cataract controls). Second, gender and age were matched between cases and controls in this study, while there were significant differences in gender and age between cases and controls in the study by Li et al. Third, healthy blood donors were used as controls in this study, while cataract patients were used as controls in the study by Li et al. [5]. Further studies are required to clarify whether IL-6 is involved in the inflammatory response in PSS.

TNF-α has the biological effects of activating lymphocytes, chemoattracting leukocytes to inflammatory tissues and inducing the production of IL-1, IL-6, nitric oxide and prostaglandins (Fig 1). Xu et al. reported that aqueous and serum TNF-α levels peaked at the early stage of inflammatory response in uveitis animal models, indicating that TNF-α may be an important cytokine in triggering the early inflammatory response of uveitis [25]. However, Zhang et al. reported that the inhibition of serum TNF-α concentration in uveitis animal models led to exacerbation of the inflammation response of uveitis [26]. In this study, we found lower serum TNF-α levels in PSS patients than normal controls although this association did not survive the Bonferroni correction for multiple comparisons (Table 3). Similarly, Li et al. found significant lower aqueous TNF-α levels in PSS patients than cataract controls [5]. These results suggest that TNF-α may not be the main cytokine in triggering the inflammatory response of PSS. Alternatively, low TNF-α levels might promote or worsen the inflammatory response of PSS. It has been reported that serum TNF-α levels are significantly reduced in patients with POAG [12], but increased aqueous TNF-α levels have also been reported in patients with uveitic glaucoma [27]. Further studies are required to elucidate whether the decrease of serum TNF-α concentration in PSS patients is associated with the characteristics of open angle glaucoma in PSS.

IFN-γ can produce cytokines such as TNF-α and IL-1β by activating macrophages, release reactive oxygen species and nitric oxide (Fig 1). Grajewski et al. reported that the genetic loss or reduction of IFN-γ could lead to increased inflammation of autoimmune uveitis in animal models [28], indicating that IFN-γ may play a protective role in the inflammation response of uveitis. In this study, we did not find significant difference in serum IFN-γ levels between PSS patients and normal controls (Table 3), consistent with the results of no significant difference in aqueous IFN-γ levels between PSS patients and cataract controls in a study by Li et al. [5]. These findings indicate that IFN-γ may not be a major cytokine inducing inflammatory response in PSS.

IL-17, mainly secreted by Th17 cells, is an early promoter of inflammatory response (Fig 1). Luger et al. reported that in the absence of IFN-γ, IL-17 alone can induce autoimmune uveitis [29]. Amadi et al. reported that an increase in IL-17 levels in peripheral blood can increase the TNF-α level in the retina in animal models of autoimmune uveitis, while a decrease in IL-17 levels can significantly reduce the inflammatory response in animal models of autoimmune uveitis [30], indicating that IL-17 is involved in the pathogenesis of autoimmune uveitis. Intriguingly, in addition to a strong pro-inflammatory effect, IL-17 has also been shown to have an anti-inflammatory effect [31]. In this study, we found that serum IL-17 levels were not significantly different between PSS patients and normal controls (Table 3). Our findings were consistent with the results of no significant difference in aqueous IL-17 levels between PSS patients and cataract controls in a study by Li et al. [5]. These findings suggest that IL-17 might not be related to PSS.

Anti-dsDNA is a characteristic marker of systemic lupus erythematosus. Previous studies have shown that anti-dsDNA can induce IL-1β secretion and Th17 cell immune response [32,33], thereby activating the inflammatory response (Fig 1). In the present study, we found that serum anti-dsDNA positive rate was significantly higher in PSS patients than normal controls (Table 4), suggesting that anti-dsDNA may be related to the pathogenesis of PSS, and thus secretion of IL-1β and IL-17 cytokines may be increased in PSS patients. However, our results showed that serum IL-1β levels were significantly lower in PSS patients than normal controls, while serum IL-17 levels were not significantly different between PSS patients and normal controls (Table 4). To strengthen our observation, we also compared serum mean concentrations of anti-dsDNA between PSS patients and controls. We found that PSS patients had significantly higher serum mean concentration of anti-dsDNA than normal controls, but this difference did not survive the Bonferroni correction for multiple comparisons. Further studies are required to elucidate whether anti-dsDNA affects the secretion of serum cytokines in PSS.

ANA refers to the autoantibodies against all the antigens in the cell, which is of great value in further diagnosis of autoimmune diseases. ANCA is a characteristic marker of ANCA-associated systemic vasculitis, which is closely related to the damage of endothelial cells. AKA and anti-CCP are the characteristic markers of rheumatoid arthritis, which is of great significance for early diagnosis and prognosis of rheumatoid arthritis. ACA is an autoantibody against platelet and negatively charged cardiolipin on endothelial cell membrane, which is commonly observed in systemic lupus erythematosus. GPI is closely related to the development of rheumatoid arthritis and is capable of stimulating the cytokine activity of TNF-α and IL-1β (Fig 1) [34]. Other studies have shown that GPI can induce the secretion of IL-6 and IL-17 (Fig 1), thereby increasing the inflammatory response [35]. Although both GPI and anti-GPI have been detected in the sera of patients with autoimmune diseases [36], serum GPI has been proposed to be a valid biomarker which is more specific to autoimmune diseases than anti-GPI [37]. As a result, we measured the serum level of GPI rather than the level of anti-GPI in this study. The results of this study showed that serum ANA, ANCA, AKA, ACA, GPI and anti-CCP positive rates were not significantly different between PSS patients and normal controls (Table 4). In a preliminary study, Shen et al. also reported that the ACA positive rate was not significantly different between PSS patients and controls although the sample sizes were small (12 PSS patients and 15 normal controls) [7]. These findings suggest that ANA, ANCA, AKA, ACA, GPI and anti-CCP may not be major contributors to the pathogenesis of PSS.

## Conclusions

In summary, we found that serum levels of IL-1β, IL-12 and IL-6 were significantly lower in PSS patients than normal controls, while there was no significant difference in serum levels of TNF-α, IFN-γ and IL-17 between the PSS and control groups. Positive rate of anti-dsDNA was significantly higher in PSS patients than the control group, while positive rates of serum ANA, AKA, ANCA, ACA, GPI and anti-CCP were not significantly different between the two groups. These results suggest that anti-dsDNA may contribute to the pathogenesis of PSS, while Th1 and Th17 related cytokines and other autoantibodies may not be major contributors to PSS. Further studies are required to elucidate the roles of these cytokines and autoantibodies in PSS.

## Supporting information

S1 Table Age, sex, IOP, Th1 and Th17 related cytokines and autoantibodies in individual PSS patients and controls. Abbreviation: PSS, Posner-Schlossman syndrome; IOP, intraocular pressure.
(XLSX)
